# Does the Concept of the “Flipped Classroom” Extend to the Emergency Medicine Clinical Clerkship?

**DOI:** 10.5811/westjem.2015.9.27256

**Published:** 2015-10-22

**Authors:** Corey Heitz, Melanie Prusakowski, George Willis, Christopher Franck

**Affiliations:** * Virginia Tech Carilion School of Medicine, Department of Emergency Medicine, Roanoke, Virginia; † University of Maryland College of Medicine, Department of Emergency Medicine, Baltimore, Maryland; ‡ Virginia Polytechnic Institute and State University, Department of Statistics, Blacksburg, Virginia

## Abstract

**Introduction:**

Linking educational objectives and clinical learning during clerkships can be difficult. Clinical shifts during emergency medicine (EM) clerkships provide a wide variety of experiences, some of which may not be relevant to recommended educational objectives. Students can be directed to standardize their clinical experiences, and this improves performance on examinations. We hypothesized that applying a “flipped classroom” model to the clinical clerkship would improve performance on multiple-choice testing when compared to standard learning.

**Methods:**

Students at two institutions were randomized to complete two of four selected EM clerkship topics in a “flipped fashion,” and two others in a standard fashion. For flipped topics, students were directed to complete chief complaint-based asynchronous modules prior to a shift, during which they were directed to focus on the chief complaint. For the other two topics, modules were to be performed at the students’ discretion, and shifts would not have a theme. At the end of the four-week clerkship, a 40-question multiple-choice examination was administered with 10 questions per topic. We compared performance on flipped topics with those performed in standard fashion. Students were surveyed on perceived effectiveness, ability to follow the protocol, and willingness of preceptors to allow a chief-complaint focus.

**Results:**

Sixty-nine students participated; examination scores for 56 were available for analysis. For the primary outcome, no difference was seen between the flipped method and standard (p=0.494.) A mixed model approach showed no effect of flipped status, protocol adherence, or site of rotation on the primary outcome of exam scores. Students rated the concept of the flipped clerkship highly (3.48/5). Almost one third (31.1%) of students stated that they were unable to adhere to the protocol.

**Conclusion:**

Preparation for a clinical shift with pre-assigned, web-based learning modules followed by an attempt at chief-complaint-focused learning during a shift did not result in improvements in performance on a multiple-choice assessment of knowledge; however, one third of participants did not adhere strictly to the protocol. Future investigations should ensure performance of pre-assigned learning as well as clinical experiences, and consider alternate measures of knowledge.

## INTRODUCTION

Emergency medicine (EM) provides students with the opportunity to care for undifferentiated patients, but the unscheduled and acute nature of the specialty makes it difficult to standardize student experiences.^1^ The variety of learning styles and differences in both medical knowledge and level of motivation among medical students in a mandatory clerkship are further complicated by varying patient chief complaints, levels of patient acuity, opportunities for procedures, and attending management styles. Thus, linking educational objectives and clinical learning during clerkships can be difficult.

The Clerkship Directors in EM (CDEM) created a set of recommended objectives and curricular goals for a required fourth-year EM clerkship in an attempt to standardize EM clerkships nationally.^2^,^3^ Educators in EM historically have created uniform didactics in an attempt to standardize the medical knowledge imparted in clerkships.^4^ With the understanding that there is a body of knowledge needing to be gained despite clinical variability and the unscheduled nature of emergency patient visits, some educators have employed asynchronous learning activities to which the students have access between shifts.^5^,^6^ This concept essentially employs the objectives of pre-learning^7^ and the exposure to standardized learning materials, such as computer-based learning modules, pre-recorded didactic lectures, selected literature, and preferred free open-access medical education materials.^6^

Adult learning theory places heavy emphasis on the applicability of gained knowledge.^8^ The concept of “inverted” or “flipped” education was developed to enhance education application. The “flipped classroom” relies on technology or other methods of information dissemination to introduce students to course content outside of the classroom so they can employ, apply or engage that information more deeply inside the classroom.^9^

During clinical clerkships, the opportunity to apply medical knowledge occurs during time in the wards, clinic, operating room, or emergency department (ED).^3^ One of the overarching objectives of the EM clerkship is to provide students with the ability to manage the undifferentiated patient.^2^,^3^ Previous authors have demonstrated that exposure to a favorable patient mix coincides with increased confidence in managing the undifferentiated patient.^10^ The challenge remains providing EM students with at least a minimum standardized exposure to high yield chief complaints that are expected to be encountered during an EM rotation and are tested in the standardized clerkship written examination.^11^ Prior works show that students can be directed to standardize their clinical experiences^12^ and that limiting the scope from one that is unfocused and unpredictable to one that creates areas of concentration makes it easier to focus on specified learning objectives, and this improves performance on examinations.^13^

We attempted to combine the experience of standardized, technology-assisted pre-learning with the previously-described quasi-standardized clinical experience to create a “flipped clerkship.” The objectives were to create an educational method in which students were directed to learn a topic prior to an assigned shift and then focus on patients with that chief complaint during their shift. We hypothesized that this unique model for directing both asynchronous learning and the clinical experiences in the ED would result in improved medical knowledge as measured by a series of multiple-choice questions.

## METHODS

### Study Setting and Participants

This was a multicenter study conducted at two academic sites, Virginia Tech Carilion School of Medicine and the University of Maryland School of Medicine, between July 1, 2013 and June 30, 2014. The study participants were either late third-year or fourth-year medical students enrolled in the required EM rotation or the EM elective at either site. All participants underwent informed consent at the beginning of the rotation, which included the assurance that participation in the study was inconsequential to their final grade on the rotation.

### Study Protocol

Study participants were randomly designated a study number that assigned them to a combination of two chief complaints commonly seen in the ED setting. The chief complaints were chest pain (CP), abdominal pain, (AP) dyspnea (SOB), and altered mental status (AMS). Once the participants were assigned to one of the six combinations of chief complaints (e.g., CP+SOB), they were told to choose two shifts during their rotation that would be “themed” shifts.

During themed shifts, participants would focus their attention on evaluating and managing patients who presented to the ED with the assigned chief complaint for that themed shift, with a goal of evaluating at least three patients with that chief complaint. They were instructed to select one shift for each of their two chief complaints to be the themed shift. Prior to the themed shift, participants were instructed to complete an interactive computer-based learning module discussing the chief complaint assigned for the themed shift. These web-based modules taught the subject material for how to evaluate patients with each of the chief complaints. These modules consisted of lecture material, web-based reading material, and questions specific to the chief complaint and were based heavily on the curriculum recommended by the CDEM and found at www.cdemcurriculum.org.^2^ All of the participants were given access to all of the learning modules at the beginning and were permitted to use them for learning purposes at any point during their rotation. The participants were instructed to perform the learning modules for the other two chief complaints (which they were not assigned to be “themed”) at a time of their choosing during the rotation.

At the end of the four-week rotation, the participants were asked to complete a 40-question examination (Appendix 1). The examination contained 10 peer-reviewed multiple-choice questions for each of the four chief complaints; students were required to answer all 40 questions (20 from their assigned themed topics, 20 from standard). Participants’ performance on the examination was not considered toward their final grade on the rotation. The examination was administered through www.saemtests.org using Logic eXtension Resourses 6.0 (LXR; Applied Measurement Professionals, Inc., Georgetown, SC) to simplify administration and tallying of results. All participants took the same examination, although the order of questions was altered by the testing software to minimize chances of unethical behavior.

After the examination, participants were asked to complete a survey. The survey asked participants to evaluate several aspects of the flipped classroom technique on a five-point scale. Points of evaluation included comparisons between the flipped clerkship and traditional learning modalities, ability to focus on the chief complaint during themed shifts, willingness of faculty and residents to allow participants to adhere to protocol, and how closely participants stuck to the protocol.

### Data Analysis

Our primary analysis was to compare scores for a flipped clerkship versus standard learning. To determine whether any observable score differences were attributable to location, site (VTC/UMD) was modeled along other predictors in a mixed model framework for both the primary (flip versus standard) and secondary (topic comparisons) analyses. The mixed model approach considers observed scores as a function of flipped status, topic, and location while modeling variability among individual students as a random effect. We removed site from analyses in which it did not exhibit a statistical association with score after accounting for other model terms.

For the primary analysis, site did not show a significant association, and therefore the comparison between flipped and standard scores was accomplished using a paired t test, which assessed flipped status in a manner equivalent to the mixed model by differencing scores between flipped and standard topics for each student. We computed group means, the t statistic, p-value, and a confidence interval for the primary analysis. The distribution of the differences was assessed graphically for normality to ensure the appropriateness of this test.

For the secondary analysis, we used the mixed model approach to compare scores among topics CP, SOB, AB, and AMS. Normality of the residuals was confirmed graphically. Topic means, p-values comparing topics, and 95% confidence intervals were computed. To determine whether the flipped approach benefitted certain topics more than others, we included a statistical interaction effect between topic and flipped status in the mixed model. Observed p-values below α=0.05 are described as statistically significant in this report.

Question performance is reported as pdiff (a measure of the difficulty of the question, with 1 signifying 100% of students answering correctly) and point biserial correlation (rpb, a measurement of the ability of a question to discriminate between high overall examinees and lower overall examinees. A higher rpb is ideal and a negative rpb signifies a flawed question.)

This study was approved by the Carilion Clinic and University of Maryland Institutional Review Boards.

## RESULTS

Sixty-nine students participated in the protocol. Examination scores were missing for 12 students who did not take the examination, and one data set had a missing participant identification number. Data for 56 students were included in the analysis. Twenty-one participants were fourth-year medical students on an elective EM rotation; 35 were students on a required EM rotation. Of these 35, 11 were late third-year students. Rotation length was four weeks for all students.

### Overall Flipped vs. Standard Score Comparison

Each student answered 20 questions on topics they were assigned to flip, and 20 questions on topics that they prepared for in the standard fashion. Site of rotation exhibited no association with scores (p=0.3861), so the paired t test was used. We saw no statistical difference when comparing scores on flipped topics vs standard topics. The mean flipped score was 14.14, and the mean standard score was 13.89 (t=−0.69 on 55 df, p=0.494, 95% CI of difference: −0.98 to 0.48). When performing the primary analysis (overall flipped vs standard score) at each participating institution individually, no difference was found (VTC: 36 students, p=0.8959; UMD: 20 students, p=0.3927). When including data from only the 28 students who replied that they followed the protocol, there was not a statistical difference between flipped and standard scores (p=0.8071). We saw no statistical difference when comparing students on required compared to elective rotations.

### Topic Comparisons

Site did not have a statistical association with score in the mixed model (p=0.3835). Statistical differences were observed between scores on the four topics. Noting that there were 10 points available per topic, the mean topic scores were AP: 7.14, AMS: 6.77, CP: 7.68, and SOB: 6.45. Scores on CP were statistically higher than the other three topics (CP:SOB, p<0.0001, 95% CI [0.76 to 1.70]; CP:AB, p=0.0251, 95% CI [−1.00 to −0.07]; CP:AMS, p=0.0002, 95% CI [−1.38 to −0.44]), and scores on AB were higher than on SOB but not AMS (AB:SOB, p=0.0038, 95% CI [0.23 to 1.16]; AB:AMS, p=0.1154, 95% CI [−0.09 to 0.84]). Scores on AMS and SOB did not significantly differ (p=0.1768, 95% CI [−0.15 to 0.79]).

### Flip Benefit for Certain Criteria

No topic benefited from being flipped when compared to the other topics. This was assessed using a statistical test for interaction between flipped status and topic (p=0.167).

### Question Performance

Average pdiff for examination questions was 0.69. Average rpb for examination questions was 0.37.

### Student Feedback

Forty-five students completed a feedback survey at the end of the rotation. Students were asked to rate the flipped method as a learning tool compared to standard; the average rating was 3.48 out of 5 with 1 being “poor/worse” and 5 being “excellent/better.” In addition, they were asked to rate their ability to evaluate patients with the assigned chief complaints, i.e., their ability to focus their shifts, with a rating of 2.66 out of 4. Most (68.9%) of respondents answered that they followed the protocol, and 31.1% responded that were not able to. The most common responses to a follow-up question of “why did you not follow the protocol?” included forgetting at the time of the shift and an inability to see patients having the chief complaint on which they were supposed to focus during that shift.

## DISCUSSION

Our results suggest that numerous challenges exist with asynchronous, targeted clinical learning in the EM student clerkship. Students participating in our flipped clerkship did not show improvement on learning, as measured by multiple-choice questioning, for specific EM topics performed in a targeted fashion when compared with those who did not similarly target their clinical learning.

Prior studies have shown both a benefit in medical education from asynchronous learning, as well as areas in which the methods did not result in differences when compared with traditional learning.^13^ Much of this research has been performed in the didactic setting, and most studies look at short-term, pre-test and post-test performance; few studies address retention. Our study assessed medium-term knowledge recall over the course of a clinical clerkship.

Clinical learning may also not fully match what students learn from textbooks or other standardized learning material, and this could have an effect on improvements in, and measurement of, knowledge. The clinical environment is variable, and students are exposed to variations in care.^1^,^12^ The effects of standardizing clinical exposure and assigning reading has had differing effects on knowledge-based assessment and clinical performance.^13^,^15^ The implications of this may be that providing a targeted, “flipped classroom” style educational approach in the clinical setting may be difficult given the unclear connection between clinical learning and knowledge gains as measured by examinations.

## LIMITATIONS

A large portion (>30%) of students did not fully engage in a themed shift or reported they did not complete their asynchronous pre-learning as per the protocol. We did not collect detailed data regarding which part of the protocol was violated. From comments provided, some students did not complete the pre-shift learning, others were unable to focus on the chief complaint during a shift, and some performed more “themed” shifts than were assigned, but the proportions of each are not known. It is unclear what effect this had on the outcomes, but the concept of pre-assigned asynchronous learning and its potential benefits is directly related to the expectation that learners complete the assignments prior to the learning session.

The clinical environment poses distinct challenges. In many flipped classroom settings, the instructor is able to control the in-classroom learning session, but this may not be possible in the clinical setting. While we encouraged students to focus on topically-appropriate patients in themed shifts, we did not keep track of their patients, and students ranked the challenge of focusing on themed topics as the greatest challenge on the feedback survey. It was our hope that the act of focused learning for the purposes of preparing for an upcoming shift would fulfill the adult learning principles of creating relevancy and goal-orientation, and therefore increase learning potential. It is possible that without the reinforcement of using the information learned (i.e., seeing patients with that chief complaint), that there is little to no benefit to prior preparation.

In addition, there was no attempt to regulate when the learners performed the modules, how much effort was put into them, or what sort of education took place during their shifts. There may have been significant heterogeneity in effort and timing of module completion; for instance, some students may have performed the module the night before a shift, while others performed it several days prior. Anecdotally, the modules take up to two hours to complete, but some students may have spent significantly less time and effort, thereby affecting their efficacy. While this unscheduled aspect of asynchronous learning is one of its inherent benefits, it may also contribute to inconsistent outcomes.

At least one student noted in written feedback that he felt the method to be useful, and therefore performed all of his shifts in a “themed” fashion. If other students similarly extended the use of themed shifts to unassigned chief complaints, this may have contributed to the lack of difference in outcomes.

The possibility of Type II error exists; the pooled standard deviation between the flipped and standard scores was 2.72, the intraclass correlation for this analysis was 0.38, and the difference in scores between the flipped and standard settings was 0.25 points. If this combination of means, standard deviations, and associations were the true state of the universe, then it would take n=743 study participants to declare statistical significance between the interventions. However, the question would remain as to what size difference would reflect a true knowledge difference.

Finally, the assessment used was a non-standardized, small set of multiple-choice questions. Multiple-choice questions may not be the ideal way to assess clinical learning,^16^ and while attempts were made to ensure question validity by expert consensus, the average difficulty was fairly low and several questions were very difficult (pdiff <0.5). However, all questions had good discriminatory value. Future work may include mixed methodology including qualitative methods and could compare entire rotation blocks performed in “flipped” fashion to those performed in standard fashion, allowing several measures of overall clerkship performance to be assessed.

## CONCLUSION

Preparation for a clinical shift with pre-assigned, web-based learning modules followed by an attempt at chief complaint-focused learning during a shift did not result in improvements in performance on a multiple-choice assessment of knowledge; however, one third of participants did not adhere strictly to the protocol. Future investigations should ensure performance of pre-assigned learning as well as clinical experiences, and consider alternate measures of knowledge.

## Supplementary Information


