# Supplementary material for: Does the Concept of the “Flipped Classroom” Extend to the Emergency Medicine Clinical Clerkship?
Source: West J Emerg Med. 2015 Oct 22;16(6):851–5. doi: 10.5811/westjem.2015.9.27256 (PMC4651581; doi:10.5811/westjem.2015.9.27256)
Supplement: Supplementary file 1 [file wjem-16-851-s001.pdf]

Abd Pain

1. A 65yo male with a history of hypertension presents to the ED after fainting during a graduation ceremony. The patient complains of acute, constant stomach pain radiating into his sides and groin along with dizziness and nausea. Vital signs on exam are BP 80/50, P120, R24, T99.0, SpO2 97% on RA.

What is the most appropriate initial study to rule out or confirm your suspected diagnosis?

Immediate angiography
Bedside abdominal ultrasound
CT abd with contrast
Electrocardiogram (EKG)
Urinalysis

2. A 30yo male presents to the ED with a several-hour history of severe abdominal pain. The pain initially presented around the belly-button but has since migrated to the right lower quadrant (RLQ). He also complains of nausea and several episodes of vomiting. He has no other medical issues. VS: 102F, BP 125/80, 100, R20. On physical exam, there is tenderness to palpation in the RLQ and firmness across the abdomen in the absence of the patient tensing his abdominal muscles. U/A is negative. Labs show WBC 18 w/88% neutrophils.

Which of the following is the most predictive of your suspected diagnosis?

Anorexia
Elevated WBCs
Migration of pain
RLQ pain
Vomiting

3. A 45yo female presents to the ED with a 6-month history of recurrent RUQ pain after meals. She is overweight and takes lisinopril. Vital signs are stable and her physical exam shows mild tenderness to deep palpation of the RUQ. A bedside ultrasound of the abdomen shows three gallstones, the largest measuring 8mm in diameter, no pericholecystic fluid, and a normal gallbladder wall.

What is the most appropriate step in management?

Analgesics, dietary changes, and outpatient follow up
Antibiotics and bowel rest (NPO overnight)

CT scan to search for alternate causes of pain
General surgery consultation in the ED

4. A 55yo male presents to the ED with a three-day history of abdominal pain. The pain is described as migratory and sporadic in waves. He has nausea and has vomited several times over the last few hours. Additionally, he has not had a bowel movement or passed gas for the past three days. On physical exam, his abdomen is distended and diffusely tender to palpation. Bowel sounds are present and are hyperechoic with the pain. Vital signs are T: 101F, BP110/80, P110, R24. Labs show WBC 14, and ABG shows pH 7.25, pO2 80, pCO2 26, HCO3 16.

What is the next best step in management?

Analgesics, antiemetics, and bowel rest
Air contrast enema
Emergent colonoscopy
NG tube, IVFs, and antibiotics

5. A 78yo female presents to the ED with sudden onset, acute, generalized abdominal pain w/vomiting. PMH is significant for chronic HTN, CVA, and DM. She takes no medications. Vital signs are: T101F, BP190/110, P120 and irregular, R24. She is in significant discomfort. On physical exam, there is diffuse tenderness to palpation in the abdomen. No bowel sounds are heard. Guaiac test is positive.
Troponin is normal. Labs show WBC 18. What is the most likely etiology of her symptoms?

Atrial thrombus with embolism
Compression of the SMA
Mesenteric artery thrombus formation
Mesenteric vein thrombosis

6. A 55yo male presents to the ED with acute abdominal pain. The pain is “up near my ribs.” He vomited once, and almost immediately after a dull, aching pain spread through his entire stomach. He has had stomach pain for several months. PMH is significant for DM and HTN. He is a chronic heavy smoker and drinker. VS: 100F, BP160/90, P100, R24. On physical exam, the abdomen is diffusely tender to palpation w/o rigidity or guarding. Upright CXR is shown.

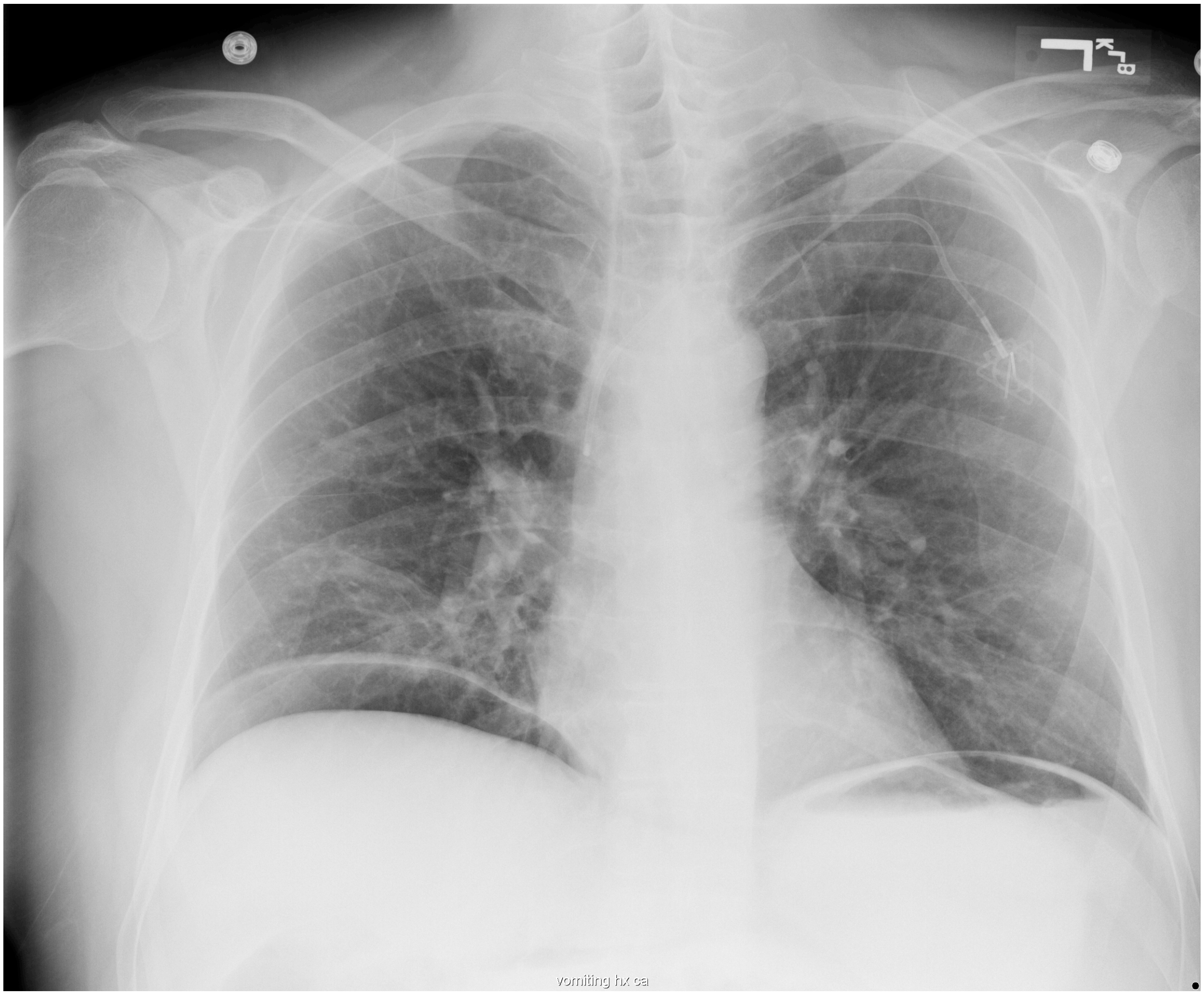

What is the most appropriate next step?

Antibiotics, IVFs, and bowel rest
CT scan of the abdomen without contrast
General surgery consultation
NG tube gastric decompression

7. A 21yo female presents to the ED with a history of right lower abdominal pain for the past several days. The pain varies between mild and intense from hour to hour. Exam shows a patient with right sided lower abdominal tenderness without rebound or guarding. Vital signs are: 98F, BP120/80, P80, R20.

What is the most appropriate initial diagnostic test?

Beta HCG
CT scan of the abdomen

Ultrasound of the right abdomen
Urinalysis

8. An 80yo female presents to the ED 30 minutes after the sudden onset of severe upper stomach pain and diaphoresis. The pain radiates to the back and both scapulae. She also complains of nausea and vomiting x 2 times. PMH is signficiant for PUD, gallstones, and stable angina. VS: 98F, BP120/70, P90, R20. Physical exam shows mild tenderness to palpation in the epigastrium with a negative Murphy's sign. Troponin levels are pending.

What is the most appropriate initial study to perform?

Abdominal plain films
Blood glucose
CT scan of the abdomen
electrocardiogam
LFTs and lipase

9. A 23yo G0 female presents to the ED with lower abdominal pain, nausea and vomiting for several days. She is sexually active and uses birth control. LMP is 13 days ago. Vital signs: 103F, BP 110/70, P110, R24. Exam shows diffuse tenderness to palpation over the lower abdomen and pelvis. A pelvic exam is performed showing greenish, purulent discharge from the cervix. The uterus and adnexa are exquisitely tender to palpation and motion without any masses noted. Cultures have been sent to the lab.

What is the most appropriate management?

Discharge pending cultures
Intramuscular ceftriazone and antiemetics
Intravenous cefoxitin and doxycycline
Oral ceftriaxone and doxycycline
Outpatient treatment with metronidazole and ampicillin

10. A 24 yo G1P1 female with no prior medical history presents with 1 hour of sudden, severe right lower quadrant abdominal pain. She is sexually active and is due to start her period this week. She takes no medicines. Vitals show HR 113, BP 123/87, RR 23, T 98.6, SpO2 99% on room air. On exam she is in severe pain, curled up on the bed. Abdomen is tender in the lower right and suprapubic with no rebound or guarding. A pregnancy test is done, and she is not pregnant. What is the most appropriate diagnostic study?

Acute abdominal series

CT scan of the abdomen and pelvis
Complete blood count with differential
Transvaginal ultrasonography
Ultrasound of the right upper quadrant

AMS

11. A 20yo female presents to the ED from college with confusion and disorientation. According to friends, the patient has been ill with vomiting and abdominal pain for 24 hours. Prior to onset of the vomiting, she had been very thirsty and losing weight for weeks. Her vital signs are 100/56, P120, T98.4, R28. Pulse oximetry is 94% on room air. On exam, she is barely arousable but is able to move everything on neurological testing.

What is the most appropriate initial test?

CT scan of the head
Blood glucose
Electrocardiogram
Serum sodium level
Urine pregnancy

12. A 40yo female postal worker presents to the ED after co-workers found her confused and disoriented in her vehicle. According to them, the patient had spent the morning delivering letters. She had just returned to the job after knee surgery. The patient takes methimazole. VS BP130/90, P120, T105F, R22, SpO2 98% RA. On physical exam, her skin is warm and dry, pupils are constricted but symmetric and reactive, and neuro exam is normal except for inability to converse or follow commands.

What is the most appropriate initial treatment for her condition?

Aspirin, IVFs
Dantrolene
Methimazole
Propylthiouricil

13. A 78 year old female is brought to the ED confused and vomiting. She does not know where is she or what her name is. Vital signs are: HR 103, BP 156/87, RR 24, T 99.6 F, and SpO2 93% on RA. On exam she is confused, holding her head, but moving all 4 extremities. Lung sounds show mild wheezes bilaterally.

While you're taking care of her, her daughter and granddaughter come to the room after being discharged for headaches and vomiting as well.

What is the most appropriate treatment for the suspected condition?

Albuterol by nebulizer
Intravenous normal saline
Oxygen by non-rebreather
Metoclopramide and fluids

14. A 45 year old alcoholic presents to the ED intoxicated after being found lying on the ground outside a convenience store. He is given IV fluids, a meal, and allowed to sober up. On recheck several hours later, he is still confused and slurring his speech. Vital signs are: HR 98, BP 106/53, RR 19, SpO2 95% on room air. His exam is normal except for an ataxic gait.

What is the most appropriate management course?

CT scan of the head
Electrocardiogram
Lorazepam and IVFs
Thiamine and glucose

15. A 24yo army recruit presents to the ED after collapsing at basic training. Fellow recruits claim he lost consciousness on the field, and in the ED he appears confused. No recent illnesses prior to this incident. He does take Claritin for allergies. Vital signs are: BP 90/60, HR 120, R22, T105. He is restless, pupils are reactive and symmetric to light, lung exam reveals mild expiratory wheezing, and his skin appears dry and hot. Neurologic exam is otherwise normal. U/A shows large blood on dipstick but no RBCs on microscopic analysis.

What is the most appropriate initial treatment?

Acetaminophen
Cool mist and fans
Dantrolene
Empiric antibiotics
Lorazepam

16. A 65yo male presents to the ED with sudden-onset R-sided weakness and aphasia. All symptoms began suddenly 1 hour ago. PMH is significant for DM, chronic back pain, arthritis, atrial fibrillation, and eczema. The patient is on glipizide, aspirin, cyclobenzaprine, metoprolol and topical hydrocortisone. Vital signs are: BP 220/105,

P113 and irregular, R20, T99F. Exam shows moderate aphasia, R sided facial droop and hand grip weakness.

What is the most appropriate initial management?

Aspirin
Diltiazem
Heparin
Intravenous tPA
Nicardipine

17. A 70yo male presents to the ED with acute onset fever, headache, confusion and nausea/vomiting. PMH is significant for HTN and DM. Medications include aspirin, insulin, and furosemide. Vital signs are: 103F, P110, BP 120/80, R20. Visual exam is normal. Nuchal rigidity is noted. CBC: WBC 17 with 90% neutrophils. LP is performed with a high opening pressure. CSF: Glu 20, Protein 70, WBC 5000 with 90% neutrophils.

What is the most appropriate treatment?

Acyclovir, betamethasone
Cefotetan and vancomycin
Fluconazole and dexamethasone
Vancomycin, ceftriaxone, ampicillin

18. A 23 year old male presents to the ED agitated and confused. He yells and screams and is generally uncooperative. His parents found him running around the yard this morning and called the police. On exam, he is angry and agitated with dilated pupils, tachycardia, warm, dry skin, and dry mucus membranes. Vital signs are: HR 128, BP 160/110, SpO2 95% on room air, and T 100.6 F.

What is the most appropriate initial management?

Acetaminophen
Cyproheptadine
Haloperidol
Lorazepam

19. A 35 year old male with a history of seizures on phenytoin presents to the ED unresponsive after having a seizure. He does not respond to painful stimuli and no history can be obtained. During transport, the patient received 5mg of diazepam IV. Initial vital signs are: HR 156, BP 175/98, SpO2 90% on NRB, RR 24 and T 99.3 F. On exam, the patient is unresponsive, diaphoretic, with rapid, shallow, ragged breathing and intermittent snoring respirations.

What is the most appropriate management at this time?

Esmolol
Intubation
Lorazepam
Phenytoin

20. A 65 year old female diabetic presents to the ED after an episode of unresponsiveness. Home medications include insulin, metformin, aspirin, metoprolol, and hydrochlorothiazide. On arrival she is cool to the touch, clammy, and confused. Neurologic exam shows a left sided facial droop and weakness. Vital signs are HR 45, BP 80/43, T 95.6 F, SpO2 94% on RA, and RR 10.

What is the most appropriate initial diagnostic study?

Blood glucose
Blood cultures
CT head
Troponin
Venous lactate

CP

21. A 60yo male presents to the ED with chest pain that began 2 hours prior to admission while working out on the elliptical machine. He has a history of hypertension for which he takes hydrochlorothiazide. Physical exam shows man clutching his chest, with slight diaphoresis, tachycardia and lungs that are clear throughout. EKG shows the following:

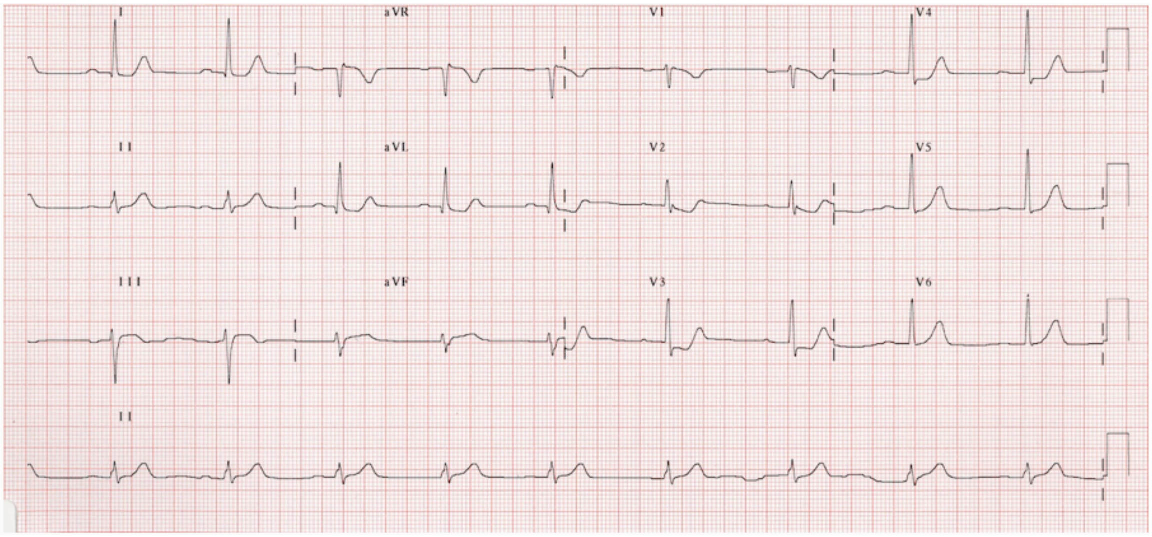

What additional testing is needed before diagnosis can be made?

a. CT scan of the chest
b. Chest xray
c. Serial cardiac markers
d. Posterior EKG leads
e. Ultrasound of the heart

22. A 75yo female comes into the ED with left sided chest pain radiating to her neck, nausea and vomiting, sweating and weakness. She also has a history of hypertension and diabetes, and it non compliant with her medications. She has no allergies. On exam she is pale, diaphoretic, and appears to be in pain. Lungs are clear and heart is regular with no murmurs. Neurologic exam is normal. Her EKG shows the following:

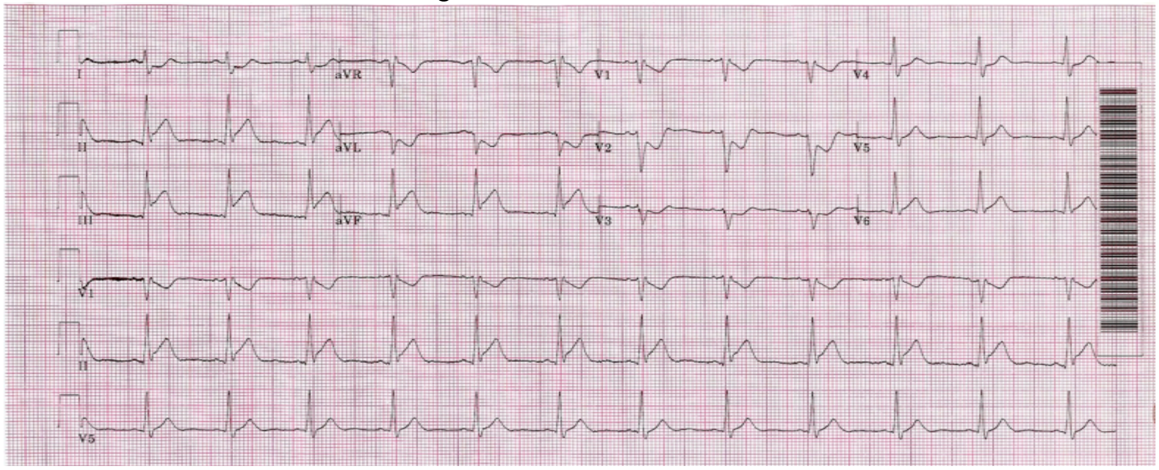

What medication has been shown to reduce mortality in her condition?

a. Acetaminophen
b. Aspirin
c. Morphine
d. Nitroglycerin
e. Oxygen

23. A 55yo male with history of CAD and hypertension presents to the ED after developing severe chest pain while taking a bowel movement. Pt states he felt an acute, popping sensation after bearing down and now with severe chest pain radiating to the neck and back. Vital signs on examination are BP 200/130, P 95, RR 25, T 98.0F, SpO2 98% on RA. What is the first step in management of this patient?

a. Cardiac markers

b. CT Angiogram of the chest
c. Chest xray
d. EKG
e. Place IV and put on monitor

24. A 55yo male with history of CAD and hypertension presents to the ED after developing severe chest pain while having a bowel movement. Pt states he felt an acute, popping sensation after bearing down and now with severe chest pain radiating to the neck and back. Vital signs on examination are BP 200/130, P 95, RR 25, T 98.0F, SpO2 98% on RA. Diminished breath sounds and hand grip strength are noted on the left side. CT Angiogram shows aortic dissection with no involvement of the ascending aorta. What is the best treatment plan?
    a. Admit to medicine for medical management
    b. Start IV esmolol, then IV nitroprusside and admit to the ICU
    c. Start IV nitroprusside, start morphine and admit to ICU
    d. Give Aspirin, start on Lovenox and admit to ICU
    e. Start PO metoprolol, nitroglycerin, monitor in the ED and discharge after blood pressure controlled

25. A 28yo male with no prior medical history comes into the ED complaining of severe, constant CP that began progressively since yesterday. States the pain is better when sitting up and is worse with movement and deep inspiration. Had recent upper respiratory infection and was given a course of Azithromycin. Physical examination shows well-developed male, no acute distress, no tenderness of anterior chest wall, RRR, no murmurs, gallops or rubs noted. No JVD, peripheral edema and lungs are clear throughout. Vital signs are: BP 135/70, P 90, RR 20, T 99.9F, SpO2 96% on RA. The following is the initial EKG for the patient:

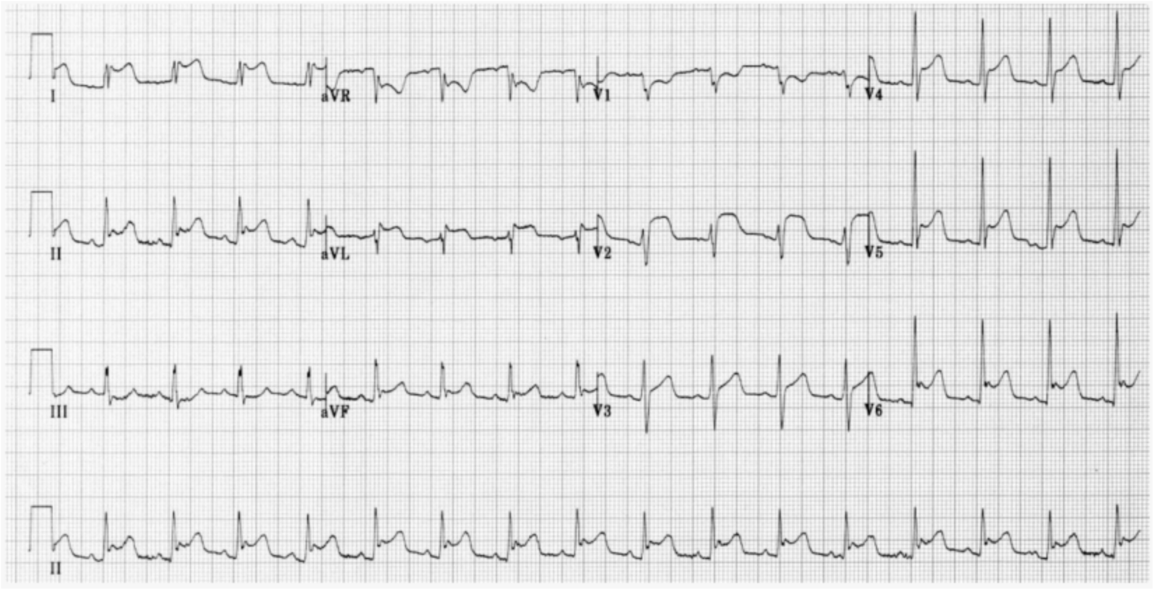

What would be an appropriate next step for this patient?

a. Re-assure patient, and discharge home with return precautions
b. Check CBC, BMP, cardiac markers, bedside echocardiography, start NSAID
c. Obtain CXR, CBC, BMP, cardiac markers and if normal discharge home with prednisone
d. Give aspirin, consult cardiology for heart catherization

26. A 36 yo female with hx of AIDS, last CD4 count 25 presents with progressive substernal chest pain, shortness of breath, subjective fevers and weight loss. The patient is not compliant with HAART therapy. Vital signs: BP 80/40, P 120, RR 25, T 99.5F, SpO2 – 93% on RA. Physical examination shows cachectic female, older than stated age, notable for JVD, tachypnea and sitting upright in bed. Heart sounds are distant. Despite receiving 2 liters of fluid the patient remains tachycardic and hypotensive. She is now becoming somnolent and her BP is 70/30. Her EKG and cardiac echo are as follows:

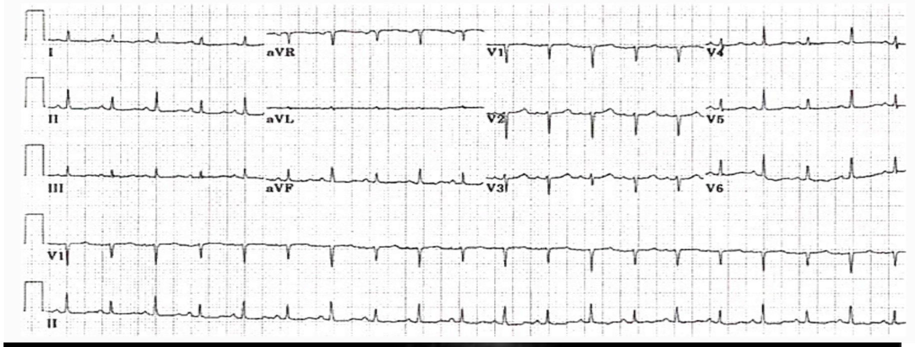

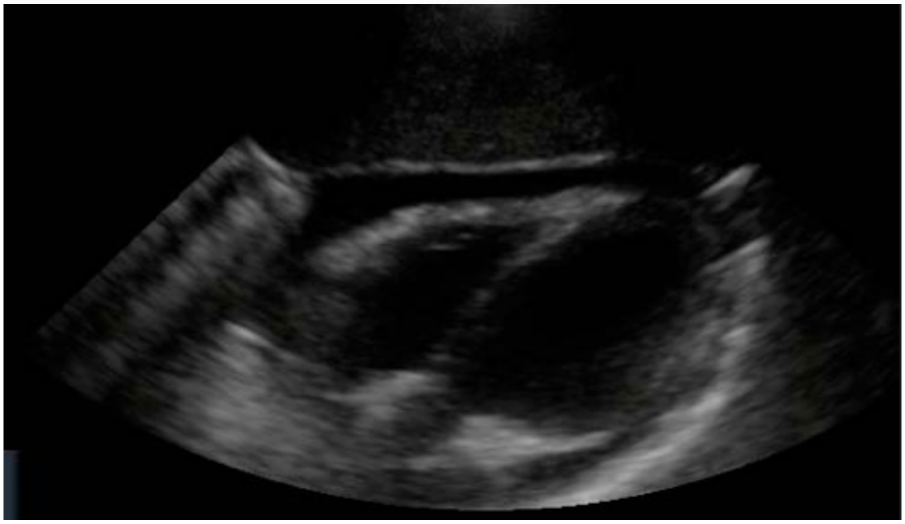

What is the next step in patient management?

a. Give Aspirin
b. Start epinephrine drip
c. Place a chest tube
d. Perform an urgent pericardiocentesis

27. A 45 yo male with no past medical history brought in by ambulance complains of moderate, substernal chest pain that began twenty minutes after snorting cocaine. Pt denies other complaints and has no allergies to medications. He received aspirin and lorazepam by the paramedics en route to the hospital. His exam is normal except that he does appear to be in discomfort and is diaphoretic.

Vitals are HR 95, BP 193/89, SpO2 98% on RA, Temp 99.2. An initial EKG shows the following:

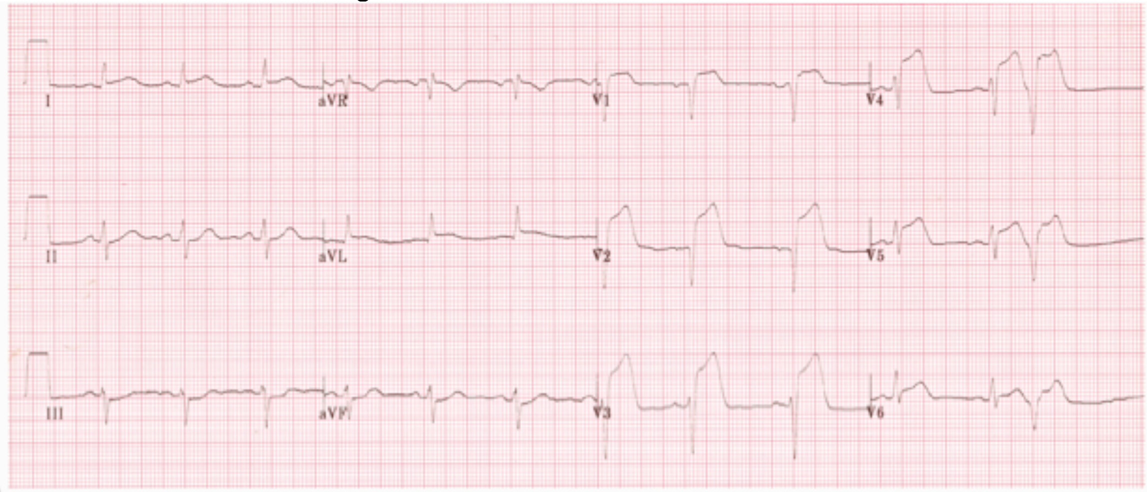

What is the most appropriate management?

- **a. Activate the cath lab**
- b. Draw cardiac markers, CBC, BMP and observe
- c. Give antinflammatories for pain
- d. Reassure patient and discharge home
- e. Give metoprolol, morphine and heparin and admit for medical management

28. A 27-year-old man previously healthy man taking no medications is brought to the emergency department by an ambulance after he was in a one-car motor vehicle collision. Paramedics report that the patient was the unrestrained driver of a car that struck a telephone pole head-on. The patient is immobilized with a cervical collar and on a board. He is conscious and says he has chest pain. Pulse rate is 140/min, respirations are 40/min, and systolic blood pressure is 80 mmHg. On physical examination, the neck veins are distended, the trachea is displaced to the right, and the left side of the chest is hyper-resonant to percussion. Heart sounds are distant. On the basis of these findings, this patient has most likely sustained which of the following traumatic injuries?
    - a. Cardiac contusion
    - b. Flail chest
    - **c. Pneumothorax**
    - d. Traumatic pericardial effusion
    - e. Rupture of the left main bronchus

29. A local gymnast comes in complaining of severe epigastric/lower chest pain that began one hour after forced vomiting. She confides in you that she has bulemia and was stressed about an upcoming competition. She takes antianxiety

medications intermittently but has no other medical problems. Per the patient she had eaten a whole pizza, prior to vomiting. Physical examination vital signs are: BP 110/50, P 115, RR 25, T – 99.1F, SpO2 93% on RA. Physical examination reveals a tearful young adult, with crepitus noted at neck region and decreased breath sounds on the left side. Pt has 3-word dyspnea and tachycardia, but no murmurs, gallops or rubs. CXR shows a left sided pleural effusion and small pneumothorax. What is the next initial diagnostic test that could help you confirm the diagnosis?

a. **Contrast esophagogram**
b. CT with contrast of the chest
c. Repeat chest xray in 6 hours
d. Transthoracic ultrasound
e. Ventilation/perfusion scan

30. A 69-year-old woman was brought to the emergency department 45 minutes after she had sudden onset of dyspnea and severe left sided chest pain. The patient was recently in a car accident and had surgery to repair a broken femur three weeks ago. Vital signs shows: BP 100/45, P 130, RR 24, T 98.9F, SpO2 88% on RA. The patient seems anxious and has two-word dyspnea. Physical examination of the chest shows accentuation of the pulmonary component of S2, unilateral crackles, and wheezing. Which of the following is most likely to confirm your suspected diagnosis?

a. 12-lead electrocardiogram
b. **CT of the chest with IV contrast**
c. Whole blood d-dimer
d. Transthoracic echocardiogram
e. Supine chest radiography

SOB

31. A 55 yo woman comes with severe bleeding from her mouth and nose. She reports increased cough, shortness of breath, and abdominal pain for 2 days. Yesterday her stool was black and she woke with "blood all over my pillow." She is pale with shallow, labored respirations. She coughs up blood, but is otherwise hard to arouse. You assist her breathing with bag-valve-mask ventilation at a rate of 30. Her heart rate is 140, BP is 82/51, and oxygen saturation is 88%. IV access is established and a NS bolus is being given. Which of the following should be done FIRST?

a. Place a nasogastric tube to check for blood in the stomach
b. Order a blood transfusion
c. **Endotracheal intubation**
d. Arrange for endoscopy

e. Arrange for pulmonary arty embolization

32. A 35-year-old man with cystic fibrosis comes complaining of sudden onset of chest pain referred to the shoulder. His pain is worse with deep breaths. He denies trauma, drug use, or palpitations. The likely treatment for this patient will include:
   a. Proton pump inhibitor
   b. Sublingual nitroglycerine
   c. Intubation
   d. Bronchoscopy
   e. **Tube thoracostomy (chest tube)**

33. A 28yo man arrives with shortness of breath and wheezing that is worsening over 2 days despite use of albuterol every 4 hours. His temperature is 38.5C, his RR is 40, and his oxygen saturation is 93% on RA. His lung examination reveals diffuse wheezing, decreased air entry throughout, and moderate work of breathing. Your initial step in management is:
   a. Systemic corticosteroids
   b. **Repeated doses or continuous inhaled beta$_2$-agonists**
   c. Subcutaneous epinephrine
   d. Oral corticosteroids
   e. Intubation

34. A 3yo lethargic boy presents in respiratory distress. He has had several days of cough and nasal congestion and today developed persistent difficulty breathing. He has had multiple ED visits for similar symptoms. VS: 98F, P130, R30, BP 90/50, SpO2 88% on NRB. Multiple nebulized treatments of albuterol and ipratropium were administered by EMS prior to arrival without relief. On exam, there is minimal air flow to auscultation bilaterally. Which finding would be most worrisome for declining status?
   a. CXR showing hyperinflation
   b. Tachypnea
   c. Tachycardia
   d. Hypoxia
   e. **Normal PCO2 levels**

35. A 65yo female presents to the ED with acute worsening of shortness of breath and non-productive cough. Her twenty-year history of COPD has left her oxygen-dependent at home. Today she is unable to catch her breath. She took several nebulizer treatments at home with no relief. She denies fever or recent infections. VS: 99F, BP 120/80, P120, R28, O2 86% on NC. She is administered

steroids, ipratropium and albuterol in the ED without much improvement. The patient is still able to converse and does not appear confused. What is the next best step in management?

a. Increase O2
b. Decrease O2
c. Intubate and ventilate
d. **NIPPV**
e. Theophylline

36. A 38yo female presents to the ED with fever and worsening productive cough. She had been prescribed azithromycin for a week in outpatient treatment but now presents with worsening symptoms and foul-smelling sputum. She had an upper endoscopy for worsening gastritis 2 weeks ago. VS: T102F, BP 130/90, P110, R28. CXR shows a right upper lobe infiltrate. What is the best antibiotic choice?

a. Doxycycline
b. Ciprofloxacin
c. TMP-SMX
d. **Clindamycin**
e. Ampicillin

37. A 16yo male presents to the ED with fever, malaise, non-productive cough and headache for one week. He has recently also noted a skin rash. VS: 101F, BP 120/80, P90, R20. On exam, his throat is erythematous without exudates, he has no lymphadenopathy, and his chest sounds clear. CXR shows interstitial infiltrates across both lobes. Sputum gram stain shows no organisms. What is the most likely cause of the patient's symptoms?

a. S. pneumonia
b. H. influenza
c. EBV
d. Legionella
e. **Mycoplasma pneumonia**

38. A 70yo male with a history of DVT presents with anxiety, chest pain and shortness of breath. He has a history of hypertension, diabetes, and smoking and is currently on low molecular weight heparin. The patient is diaphoretic, but the chest is clear on exam. CXR shows no abnormalities. EKG shows sinus tachycardia and RBBB. Which of the following studies would most likely reveal the diagnosis?

a. Serial troponins

b. **CT angiography**
c. ECHO
d. ABG
e. BNP

39. A 70-year-old man presents with shortness of breath, fever and productive cough for 3 days. He is a chronic smoker on home O2. CXR shows right lower lobe consolidation. In the ED, his condition worsens and he requires intubation and ventilation with subclavian IV access. Immediately after that management, he deteriorates rapidly. Exam reveals absent right side breath sounds. What is the next best step in management?

a. CXR
b. ABG
c. IV fluids and pressors
d. **Needle thoracostomy**
e. Cricothyroidotomy

40. A 66-year-old woman comes with worsening shortness of breath. She reports increasing breathlessness when laying flat, swelling of her feet and ankles, and extreme shortness of breath when trying to climb stairs. She has not been able to drink much because of mild nausea. Temperature is 98.8, Respirations 42, HR 120, and oxygen saturation 93% on oxygen face mask. The best first step in management is:

a. Sublingual nitroglycerine
b. Aggressive lasix infusion
c. Intubation
d. EKG for presumed myocardial infarction
e. Tube thoracostomy for pneumothorax
